# Septic Infection from a Tooth

**Published:** 1903-11

**Authors:** James R. Piper

**Affiliations:** Boston, Mass.


### SEPTIC INFECTION FROM A TOOTH.

BY JAMES R. PIPER, D.D.S., BOSTON, MASS.

A SHORT time ago the following case came to my notice, and the results to me were little more than we usually expect to get from cases coming under the head of abscessed teeth; in fact, complications set in which seem almost impossible to connect directly with a tooth. And yet why should not we have just this sort of thing take place when favorable conditions exist?

The case referred to is as follows: March 1, Mrs. W., aged about thirty-eight, who had always enjoyed good health, first noticed a red spot on the left cheek in the region of the angle of the inferior maxillary bone. March 5 she visited her dentist and was advised by him to have the lower twelfth-year molar on the left side extracted. March 8 a second dentist was called upon, who told her the gum was so badly swollen he did not consider it wise to extract the tooth, but consented to lance the swollen parts from the inside of the cheek, telling the patient he thought her trouble was at an end. March 11, still suffering with swelling and pain, dentist No. 3 was visited, who administered gas and extracted the offending member, assuring the patient that the whole tooth had been removed. This not giving her the desired relief, she again applied to dentist No. 1, who removed what he said was a part of the tooth supposed to have been extracted by dentist No. 3. March 22 her physician made a deep incision with a bistoury through the swelling from the outside, but without giving the desired relief. April 5, the face looking so badly and the patient's physician realizing that somthing more radical must be done, he opened up the parts again, and this time maintained a drainage by means of silk ligatures, the physician making several visits a day, considering his patient in a serious condition. Under this treatment she improved until April 25, when during the evening of that day she complained of pain in her left hand. The pain grew worse, and by midnight it was so severe she was obliged to hold her hand in water as hot as the flesh would tolerate in order to get relief. She kept the hot-water treatment up until morning when the physician arrived.

A consultation was held with one of the surgeons from the Boston City Hospital. The hand was found to be badly swollen, and the arm was somewhat swollen as far up as the elbow, but above, to the patient's shoulder, it was in a normal condition. Ether was administered, the opening in the cheek enlarged, and the bone thoroughly curetted. Another opening was made in the palm of the hand, and both were dressed with lysol gauze, the dressings being changed every day until the parts healed by granulation. After the operation no pain was experienced by the patient.

The swelling of the hand was of septic origin, due, probably, to the patient having a scratch on the palm which in some way became infected from the discharge on the side of the face.

There are three points which this case brings out quite forcibly, —viz., first, do not allow such cases to run too long before operating; second, absolute cleanliness should be maintained; third, operate from the inside of the mouth.

While the case just cited happened to a person of liberal means, she could not have realized the importance of absolute cleanliness. This is not an uncommon failing among many of our best people, and we should not lose an opportunity to inform our patients of the seriousness of neglect.

